# Comparison between TG‐51 and TG‐21: Calibration of photon and electron beams in water using cylindrical chambers

**DOI:** 10.1120/jacmp.v1i3.2643

**Published:** 2000-06-01

**Authors:** S. H. Cho, J. R. Lowenstein, P. A. Balter, N. H. Wells, W. F. Hanson

**Affiliations:** ^1^ Department of Radiation Physics The University of Texas M.D. Anderson Cancer Center 1515 Holcombe Boulevard, Box 547 Houston Texas 77030

**Keywords:** beam calibration protocol, cylindrical chambers, TG‐51, TG‐21, dosimetry

## Abstract

A new calibration protocol, developed by the AAPM Task Group 51 (TG‐51) to replace the TG‐21 protocol, is based on an absorbed‐dose to water standard and calibration factor $\left(N_{D,w}\right)$, while the TG‐21 protocol is based on an exposure (or air‐kerma) standard and calibration factor $\left(N_{x}\right)$. Because of differences between these standards and the two protocols, the results of clinical reference dosimetry based on TG‐51 may be somewhat different from those based on TG‐21. The Radiological Physics Center has conducted a systematic comparison between the two protocols, in which photon and electron beam outputs following both protocols were compared under identical conditions. Cylindrical chambers used in this study were selected from the list given in the TG‐51 report, covering the majority of current manufacturers. Measured ratios between absorbed‐dose and air‐kerma calibration factors, derived from the standards traceable to the NIST, were compared with calculated values using the TG‐21 protocol. The comparison suggests that there is roughly a 1% discrepancy between measured and calculated ratios. This discrepancy may provide a reasonable measure of possible changes between the absorbed‐dose to water determined by TG‐51 and that determined by TG‐21 for photon beam calibrations. The typical change in a 6 MV photon beam calibration following the implementation of the TG‐51 protocol was about 1%, regardless of the chamber used, and the change was somewhat smaller for an 18 MV photon beam. On the other hand, the results for 9 and 16 MeV electron beams show larger changes up to 2%, perhaps because of the updated electron stopping power data used for the TG‐51 protocol, in addition to the inherent 1% discrepancy presented in the calibration factors. The results also indicate that the changes may be dependent on the electron energy.

PACS number(s): 87.66.–a, 87.53.–j

## INTRODUCTION

Recently, the AAPM Task Group 51 (TG‐51) introduced a new calibration protocol for clinical high‐energy photon and electron beams.^1^ The protocol (generally known as TG‐51) relies on an absorbed‐dose to water standard and calibration factor $\left(N_{D,w}\right)$ while its predecessor, the TG‐21 protocol,^2^ is based on an exposure (or air‐kerma) standard and calibration factor $\left(N_{x}\right)$. Also, there are some differences between these protocols in the following aspects: (a) the electron stopping‐power data, (b) the energy‐dependent correction factors, and (c) the procedures for beam calibration.^1^ ^,^ ^2^ Due to these differences, the results of clinical reference dosimetry based on TG‐51 may be somewhat different from those based on TG‐21.

The Radiological Physics Center (RPC) has conducted a systematic comparison between these two protocols, in which photon and electron outputs following both protocols were compared under identical conditions. This comparison would provide the magnitude of anticipated changes in photon and electron beam output calibrations after the implementation of the TG‐51 protocol. This study was conducted with cylindrical chambers selected from the list given in the TG‐51 report,^1^ covering the majority of current manufacturers.

## METHODS AND MATERIALS

### Basic equations and definitions

For the sake of brevity, only a brief explanation for each dosimetric parameter is provided here. More details can be found in the original task group reports.^1^ ^,^ ^2^ Note some of the notations used in the TG‐21 report are changed here for an easier comparison with those used in the TG‐51 report.

### TG‐51 protocol

In the TG‐51 protocol,^1^ the fully corrected ion chamber reading, *M*, is defined as

(1) $$M=P_{\text{ion}}P_{T,P}P_{\text{elec}}P_{\text{pol}}M_{\text{raw}},$$

where $P_{\text{ion}}$ is a correction factor to take into account the incomplete collection of charge from an ion chamber; $P_{T,P}$ is the temperature‐pressure correction factor; $P_{\text{elec}}$ is the electrometer correction factor (C/rdg); $P_{\text{pol}}$ is the polarity correction factor; and $M_{\text{raw}}$ is the uncorrected ion chamber reading at the point of measurements (*rdg*).

The beam quality (*Q*) for photon beams is defined by the following quantities:

$\%\text{dd}{\left(10\right)}_{x}$: the photon component of the percentage depth‐dose at 10 cm depth in a $10\times 10\;{\text{cm}}^{2}$ field at an SSD (source‐to‐surface distance) of 100 cm.
%dd(10): the measured percentage depth‐dose at 10 cm depth in a $10\times 10\;{\text{cm}}^{2}$ field at an SSD of 100 cm. Thus %dd(10) includes the effects of electron contamination in the beam.

For an arbitrary photon beam with a beam quality *Q*, the absorbed‐dose to water $D_{w}^{Q}$ is given by the TG‐51 report^1^ as

(2) $$D_{w}^{Q}=Mk_{Q}N_{D,w}^{60_{\text{Co}}}\;\;\;[\text{Gy}]$$

where

$k_{Q}$: the beam quality conversion factor, a chamber specific factor which accounts for the change in the absorbed‐dose to water calibration factor between the beam quality of interest, *Q*, and the beam quality for which the absorbed‐dose calibration factor applies (i.e., C60o),
ND,wC60o: the absorbed‐dose calibration factor under reference conditions in a C60o beam.

For an electron beam, the beam quality (*Q*) is represented by $R_{50}$, the depth in water in a $10\times 10\;{\text{cm}}^{2}$ or larger beam of electrons at an SSD of 100 cm at which the absorbed‐dose falls to 50% of the dose maximum.^1^ The absorbed‐dose to water $\left(D_{w}^{Q}\right)$ is given by the TG‐51 report^1^ as

(3) DwQ=MPgrQkR50′kecalND,wC60o  [Gy],

where $P_{gr}^{Q}$ is the gradient correction factor; $k_{R_{50}}^{\prime }$ is the electron quality conversion factor to convert $N_{D,w}^{Q_{\text{ecal}}}$ into $N_{D,w}^{Q}$ for any beam quality *Q*; $k_{\text{ecal}}$ is the photon‐electron conversion factor to convert ND,wC60o into an electron beam absorbed‐dose calibration factor $N_{D,w}^{Q_{\text{ecal}}}$ for a selected electron beam quality $Q_{\text{ecal}}$ (i.e., $R_{50}=7.5\;\text{cm}$). Note the product, $P_{gr}^{Q}k_{R_{50}}^{\prime }k_{\text{ecal}}$, is equivalent to $k_{Q}$ for electron beams.

### TG‐21 protocol

Following the TG‐21 protocol,^2^ the corrected ion chamber reading, *M*, can be defined as

(4) $$M=P_{\text{ion}}P_{T,P}P_{\text{elec}}M_{\text{raw}}.$$

Note that $P_{\text{pol}}$ is discussed in the protocol but not explicitly included in the equations for the TG‐21 protocol.^2^ Therefore, no polarity correction was applied for TG‐21 calculations in this study. The absorbed‐dose to water due to a photon beam is then given by

(5) $$D_{\text{water}}=MN_{\text{gas}}{\left(\frac{\bar{L}}{P}\right)}_{\text{air}}^{\text{water}}P_{\text{wall}}P_{\text{repl}}\;\;[\text{Gy}],$$

where $N_{\text{gas}}$ is the cavity‐gas calibration factor; ${\left(\bar{L}/\rho \right)}_{\text{air}}^{\text{water}}$ is the ratio of the mean, restricted, collision mass stopping power between water and air; $P_{\text{wall}}$ is the wall correction factor; $P_{\text{repl}}$ is the correction factor for replacement of the phantom material by an air cavity. The absorbed dose to water due to an electron beam is calculated by the TG‐21 protocol^2^ as

(6) $$D_{\text{water}}=MN_{\text{gas}}{\left(\frac{\bar{L}}{\rho }\right)}_{\text{air}}^{\text{water}}P_{\text{repl}}\;\;[\text{Gy}].$$

### Relation between absorbed‐dose (TG‐51) and air‐kerma (TG‐21) standards

A theoretical relationship between absorbed‐dose and air‐kerma standards (or calibration factors) can be derived by comparing the absorbed‐dose to water determined for a C60o beam using the TG‐51 and TG‐21 protocols. Using $k_{Q}=1.0$ and assuming $P_{\text{pol}}\approx 1.0$, the following relationship for a C60o beam can be obtained by taking the ratio between Eqs. (2) and (5):

(7) $$\frac{D_{w}}{D_{\text{water}}}=\frac{N_{D,w}}{N_{k}{\left(0.8791\right)}^{-1}\left(\frac{N_{\text{gas}}}{N_{x}}\right)P_{\text{repl}}P_{\text{wall}}{\left(\frac{\bar{L}}{\rho }\right)}_{\text{air}}^{\text{water}}},$$

where the value 0.8791 is the conversion factor from exposure to air‐kerma (kinetic energy released in air);^3^ $N_{k}$ and $N_{x}$ are the air‐kerma and exposure calibration factors, respectively. If no systematic difference exists in both calibration standards and protocols, Eq. (7) should be equal to unity and, as a result, the ratio, $N_{D,w}/N_{k}$, can be calculated as

(8) $${\left(\frac{N_{D,w}}{N_{k}}\right)}_{\text{calc}}={\left(0.8791\right)}^{-1}\left(\frac{N_{\text{gas}}}{N_{x}}\right)P_{repl}P_{well}{\left(\frac{\bar{L}}{\rho }\right)}_{\text{air}}^{\text{water}}.$$

Note that no measured value is necessary for Eq. (8). All the required numerical values can be obtained from the TG‐21 report.^2^ The ratio, $N_{D,w}/N_{k}$, can also be directly determined using the absorbed‐dose and air‐kerma calibration factors measured at the calibration laboratory and is denoted as ${\left(N_{D,w}/N_{k}\right)}_{\text{meas}}$ in this study.

### Measurements

All measurements were performed with selected cylindrical chambers from the list given in the TG‐51 report,^1^ in a $30\times 40\times 40\;{\text{cm}}^{3}$ water phantom. For nonwaterproof chambers, a waterproofing sleeve with a 1‐mm‐thick polymethylmethacrylate (PMMA) wall was used. The makes and models of the chambers are given in Table I. Both absorbed‐dose and air‐kerma calibration factors were obtained from the Accredited Dosimetry Calibration Laboratory (ADCL) at the M.D. Anderson Cancer Center (MDACC) based on standards traceable to the National Institute of Standards and Technology (NIST). The electrometer (Keithley model 602) was also calibrated at the MDACC ADCL. Beam outputs were determined for 6 and 18 MV photon beams, and 9 and 16 MeV electron beams from a Clinac 2100C (Varian Oncology Systems, Palo Alto, CA) following TG‐51 and TG‐21. Beams were incident on the phantom surface, vertically (i.e., at gantry 180°), at an SSD of 100 cm. All measurements were repeated at least three times to ensure the reproducibility of each measurement.

**Table I:** Makes and models of the chambers used.

Ion chamber	Serial number	Wall material	Al electrode	$N_{D,w}(10^{7}\;\text{Gy}/C)$	$N_{x}(10^{9}\;R/C)$
NEL 2571	1503	graphite	yes	4.5873	4.7331
NEL 2571	1864	graphite	yes	4.5257	4.6799
PTW N23333	1516	PMMA	yes	5.0913	5.2690
PTW N30001	1483	PMMA	yes	5.2844	5.4712
Capintec PR06C	CII.68624	C‐552	no	4.7445	4.9290
Exradin A‐12	174	C‐552	no	4.9801	5.1249

### Photon beam

The beam quality of the photon beams for the TG‐51 protocol was determined in the following manner: First, the depth of maximum ionization was searched. Then, the chamber was positioned at $\left(10\;\text{cm}+0.6r_{\text{cav}}\right)$ to determine %dd(10), where $r_{\text{cav}}$ is the radius of the air cavity in an ion chamber.^1^ For the 6 MV photon beam, %dd(10) was taken to be equal to $\%\text{dd}{\left(10\right)}_{x}$. For the 18 MV photon beam, $\%\text{dd}{\left(10\right)}_{x}$ was obtained following the procedure described in the TG‐51 report^1^ using a 1 mm lead foil located at 55 cm from the phantom. After determining the quality of the photon beams, relevant $k_{Q}$ factors were obtained from the table given in the TG‐51 report.^1^ The beam quality of the photon beams for the TG‐21 protocol^2^ was determined as the ratio of the TMRs (tissue maximum ratio) between 10 and 20 cm depths in water from the institution's clinical dosimetry data.

Calibrations were performed with the chamber center positioned at 10 cm depth in water. Readings for 200 Monitor Units (MU) were taken at three different voltage settings (i.e., –300, –150 and +300 V) to determine $P_{\text{pol}}$ and $P_{\text{ion}}$ and an appropriate correction was applied for temperature and pressure $\left(P_{T,P}\right)$. Note $P_{\text{pol}}$ for photon and electron beams was typically less than 0.2% for all the chambers used in this study. Using the corrected readings, absorbed doses at 10 cm depth were determined for TG‐51 and TG‐21 protocols using Eqs. (2) and (5), respectively.

### Electron beam

Electron beam outputs were measured at $d_{\text{ref}}$ for TG‐51 and at $d_{\max}$ for TG‐21 (Table II), respectively. The reference depth $\left(d_{\text{ref}}\right)$ and related dosimetric quantities were determined following the procedure described in the TG‐51 report,^1^ while the depth of maximum dose $\left(d_{\max}\right)$ and necessary data (e.g., $E_{0},R_{p}$, etc.) for the TG‐21 protocol^2^ were obtained from the institution's clinical dosimetry data. Note $d_{\max}$ in this study was an effective point of measurements with an appropriate shift in chamber location, as recommended by the TG‐25 report.^4^ Similar to photon beam calibration, readings for 200 MUs were taken at three different voltage settings (i.e., –300, –150 and +300 V) to determine $P_{\text{pol}}$ and $P_{\text{ion}}$ and an appropriate correction was applied for temperature and pressure $\left(P_{T,P}\right)$. For the TG‐51 protocol, additional readings were taken at $\left(d_{\text{ref}}+0.5r_{\text{cav}}\right)$ to determine the gradient correction factor $\left(P_{gr}^{Q}\right)$.^1^ Outputs at $d_{\text{ref}}$ were converted to the dose rates at $d_{\max}$ using the clinical depth dose data and compared with the values from the TG‐21 protocol.

**Table I:** $d_{\text{ref}}$ and $d_{\max}$ for electron calibration.

Electron energy (MeV)	$d_{\text{ref}}$ (cm)	$d_{\max}$ (cm)
9	2.0	2.1
16	3.8	3.4

## RESULTS

### Photon beam

To investigate the basic difference between the absorbed‐dose and air‐kerma standards (or calibration factors), the absorbed‐dose to water for a C60o beam can be determined using the TG‐51 and TG‐21 protocols. Alternatively, if a C60o beam is unavailable, the ratio between the absorbed‐dose and air‐kerma calibration factors obtained from the standards lab, i.e., ${\left(N_{D,w}/N_{k}\right)}_{\text{meas}}$, can be compared with the same ratio calculated using Eq. (8). The ratio between ${\left(N_{D,w}/N_{k}\right)}_{\text{meas}}$ and ${\left(N_{D,w}/N_{k}\right)}_{\text{calc}}$ then yields the difference in the absorbed‐dose to water for a C60o beam following the TG‐51 and TG‐21 protocols. Table III shows the ratio between the measured and calculated ratios. The discrepancy between measured and calculated values is about 1% for all chambers tested. This means that the absorbed‐dose to water for a C60o beam determined by the TG‐51 protocol will be about 1% higher than that determined by the TG‐21 protocol.

**Table III:** Comparison between absorbed‐dose and air‐kerma calibration factors. Measured ratios in this table are obtained using measured absorbed‐dose and air‐kerma calibration factors from the MDACC ADCL based on standards traceable to NIST. Note $N_{k}=0.8791N_{x}$ and the numerical parameters (e.g., $L/\rho ,P_{\text{wall}},P_{\text{repl}}$, etc.) for calculations using Eq. (8) are based on the TG‐21 report (Ref. 2).

Ion chamber	Serial number	$N_{D,w}/N_{k}$ (Meas.)	$N_{D,w}/N_{k}$ (Calc.)	Meas./Calc.
NEL 2571	1503	1.102	1.088	1.013
NEL 2571	1864	1.100	1.088	1.011
PTW N23333	1516	1.099	1.086	1.012
PTW N30001	1483	1.099	1.086	1.012
Capintec PR06C	CII.68624	1.095	1.079	1.015
Exradin A‐12	174	1.105	1.093	1.011

Table IV lists the ratio of absorbed‐dose to water determined by TG‐51 to that determined by TG‐21 for the 6 and 18 MV photon beams. Note the results from both NEL and PTW chambers in Table I are averaged here and the average values for a C60o beam from Table III are also included. The same 1% discrepancy observed for the C60o beam is seen for a 6 MV photon beam. The discrepancy is somewhat smaller for the 18 MV photon beam, indicating that the discrepancy might decrease with increasing photon energy. This could be partially due to some compensating effect between various dosimetric parameters in both calibration protocols.

**Table IV:** Comparison between TG‐51 and TG‐21 calibrations (photon beam). The ratios represent the comparison at 10 cm depth in water, a recommended depth for photon beam calibration in the TG‐51 protocol. Presented results have an estimated uncertainty of less than ±0.2% excluding the inherent uncertainty associated with the calibration factors.

Ion chamber	C60o (TG‐51/TG‐21)	6MV (TG‐51/TG‐21)	18 MV (TG‐51/TG‐21)
NEL 2571	1.012	1.010	1.007
PTW N23333 & N30001	1.012	1.010	1.006
Capintec PR06C	1.015	1.011	1.004
Exradin A‐12	1.011	1.008	1.002

### Electron beam

Table V lists the ratio of absorbed‐dose to water determined by TG‐51 to that determined by TG‐21 for 9 and 16 MeV electron beams. The discrepancies are somewhat larger than those for photon beams, approaching 2% at 16 MeV. Note the electron stopping power data used in TG‐51 are based on realistic clinical electron beams,^5^ whereas the data used in TG‐21 are based on mono‐energetic electrons. This may explain a somewhat larger discrepancy for electrons than that seen for photons. The results also indicate that the magnitude of changes may be dependent on the electron energy.

**Table V:** Comparison between TG‐51 and TG‐21 calibrations (electron beam). Note the ratios represent the comparison at $d_{\max}$ in water for each electron energy. Presented results have an estimated uncertainty of less than ±0.2% excluding the inherent uncertainty associated with the calibration factors.

Ion chamber	9 MeV (TG‐51/TG‐21)	16 MeV (TG‐51/TG‐21)
NEL 2571	1.015	1.021
PTW N23333 & N30001	1.014	1.017
Capintec PR06C	1.014	1.016
Exradin A‐12	1.014	1.016

## DISCUSSION

Table III suggests an approximate 1% discrepancy between the absorbed‐dose to water determined for a C60o beam by TG‐51 based on an absorbed‐dose standard and that determined by TG‐21 based on an air‐kerma (or exposure) standard. This result implies that either one of the following could be possible: (a) an inherent discrepancy in either of the standards or (b) an inaccuracy in the TG‐21 formalism for converting air‐kerma to absorbed dose. This question may be pursued, somewhat indirectly, by investigating the difference between the national standards, especially between the US and Canada where the same calibration protocol is being used. The US standards lab (NIST) and the Canadian standards lab (National Research Council of Canada (NRCC)) are aware of the difference, up to 1%, between their air‐kerma standards and a somewhat smaller discrepancy for their absorbed‐dose standards.^6^ ^,^ ^7^ There is also an additional uncertainty for the transfer of the standards from NIST through the ADCL to a user's chamber. Therefore, most probably, the results presented in Table III of this study would be different if the air‐kerma and absorbed‐dose calibration factors were derived from a different ADCL or from the Canadian standards. In fact, an investigation by a Canadian group shows better agreement (within 0.5%) in measured and calculated ratios between absorbed‐dose and air‐kerma calibration factors.^8^ Therefore, we may argue that a consistent 1% discrepancy in our results is mostly due to the uncertainties in the two standards, absorbed‐dose and air‐kerma.

The implication of our analysis on the nature of the discrepancy is that any user may be able to estimate the magnitude of changes resulting from the implementation of the TG‐51 protocol by performing the comparison shown in Table III of this study. Thus it is important for a first‐time‐TG‐51‐user to obtain both the absorbed‐dose and air‐kerma calibration factors on the same chamber and to perform a comparison similar to that we have made in this work. The discrepancies between TG‐51 and TG‐21 for all photon beam calibrations are not expected to be significantly larger than the difference observed from this comparison for a C60o beam. If the changes are more than expected, the users should suspect some other errors in the implementation of the TG‐51 or TG‐21 protocols. As mentioned previously, the discrepancy for electron beam calibrations is expected to be slightly larger than that for a C60o beam and the discrepancy more than 2% may still be possible. However, if the changes are excessive (>3%), the users should check other sources of error as well.

## CONCLUSIONS

Photon and electron beam outputs were measured in water following both TG‐51 and TG‐21 protocols using cylindrical chambers under identical conditions. Measured ratios between absorbed‐dose and air‐kerma calibration factors, based on the standards traceable to the NIST, were compared with calculated values based on the TG‐21 protocol. The comparison shows approximately a 1% discrepancy between measured and calculated ratios. This discrepancy may provide a reasonable measure of possible changes between the absorbed‐dose to water determined by TG‐51 and that determined by TG‐21 for photon beam calibrations. Therefore, it is recommended that a first‐time‐TG‐51‐user should obtain both the absorbed dose and air‐kerma calibration factors on the same chamber, and perform an initial comparison as described in this study to estimate the inherent discrepancy expected from the implementation of the TG‐51 protocol. The typical change in a 6 MV photon beam calibration following the implementation of the TG‐51 protocol was about 1%, regardless of the chamber used, reflecting the 1% discrepancy presented in the calibration factors. The change was somewhat smaller for an 18 MV photon beam, indicating that the magnitude of change might decrease with increasing photon energy. The changes were somewhat larger for electron beams, perhaps because of the new electron stopping power data used for the TG‐51 protocol, in addition to the inherent 1% discrepancy in the calibration factors. The electron results show changes up to 2% and also indicate that the changes may be dependent on the electron energy.

If the changes in the absorbed‐dose to water, after the implementation of the TG‐51 protocol, are more than the discrepancy in the calibration factors for photons or are more than 3% for electrons, the users should suspect some errors in their implementation of the TG‐51 protocol and may want to contact the authors for data comparison.

## ACKNOWLEDGMENTS

This investigation was supported by Public Health Service Grant No. CA 10953 awarded by the National Cancer Institute, Department of Health and Human Services. The authors thank D. W. O. Rogers at NRCC for helpful discussion.
